# Localization of a Bullet in a Firearm Injury Victim Using X-ray Imaging During Autopsy: A Case Report

**DOI:** 10.31729/jnma.8811

**Published:** 2024-11-30

**Authors:** Kaschev Shrestha, Nishana Shrestha, Salmalee Yadav, Dhirendra Yadav

**Affiliations:** 1 Department of Forensic Medicine, College of Medical Sciences, Bharatpur, Chitwan, Nepal; 2 College of Medical Sciences, Bharatpur, Chitwan, Nepal; 3 Department of Forensic Medicine, Patan Academy of Health Sciences, Lagankhel, Lalitpur, Nepal; 4 Patan Academy of Health Sciences, Lagankhel, Lalitpur, Nepal

**Keywords:** *case report*, *firearms*, *injury*, *forensic*, *xray*

## Abstract

Firearm injuries can result in varying degrees of trauma, depending on factors such as the type of firearm, range, and wound location. This case report presents the role of X-ray imaging in detecting a projectile causing fatal penetrating abdominal trauma in a 45-year-old male. The patient was found with a gunshot wound in his abdomen, and despite medical interventions, he succumbed to his injuries. X-ray imaging revealed a hyper-dense area resembling a bullet lodged in the abdominal cavity. Autopsy confirmed a close-range gunshot with significant internal damage to the peritoneum, mesentery, liver, and the bullet lodged near the L1 vertebra. This case underscores the value of radiological imaging in identifying projectile trajectory and injury severity, particularly in resource-limited settings. X-rays assist in distinguishing between penetrating and perforating wounds, aiding both forensic investigation and legal procedures. The integration of imaging in forensic autopsies improves the accuracy of injury assessment, especially in cases involving gunshot wounds.

## INTRODUCTION

Firearms can produce various degrees of injury based on type, range, number of shots, and wound location.^1^ Mainly, they can produce penetrating wounds if the projectile enters the body and remains in soft tissue compartments or perforating wounds if it finds an exit pathway out of the body.^2^ Prior to the autopsy, radiological imaging such as an X-ray remains the most basic, easily accessible, and essential method to identify possible injuries, position.^3^ Radiological imaging can also help to determine the position of the assailant in relation to the victim and differentiate whether the injury was homicidal, suicidal, or accidental.^4^ The case report is about locating a projectile causing penetrating abdominal trauma by firearm with the help of an x-ray.

## CASE REPORT

A 45-year-old male was discovered injured inside his locked room, where he was found with a firearm entry wound in his abdomen. Upon discovery, both the floor and the patient's body were observed to be soaked in blood.

At 09:40 hours, the patient was promptly transported for medical care, where preliminary radiological investigations were conducted. Unfortunately, despite efforts, the patient's condition deteriorated, and he succumbed to his injuries at 13:08 hours during treatment at Medical College.

**Figure 1:**
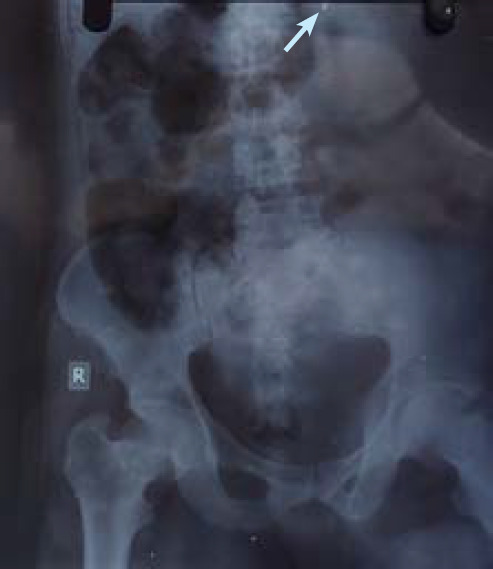
Abdominal X-ray with an anterior-posterior view revealing a hyper-dense area on the upper left side, resembling the shape of the nose of the bullet.

The abdominal X-ray with an anterior-posterior view revealed a hyper-dense area on the upper left side, resembling the shape of the nose of the bullet (Figure 1). This finding highly suggests a metallic projectile lodged within the abdominal cavity. The location of this hyper-dense area correlates with the entry wound observed on clinical examination, further supporting the diagnosis of a penetrating gunshot wound. The absence of significant fragmentation or secondary projectiles indicates a single, direct bullet trajectory consistent with the clinical presentation.

**Figure 2:**
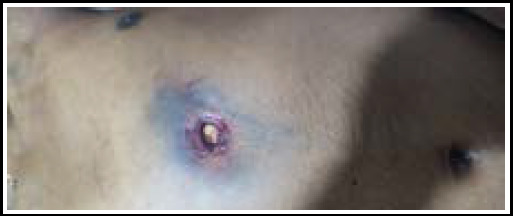
A circular entry wound located on the epigastric region of abdomen, surrounded by a reddish-brown abrasion collar.

The examination was conducted on the same day at 19:00 hours, with complete rigor mortis noted. External examination revealed a circular entry wound measuring 1 cm in diameter on the abdomen (Figure 2). Surrounding the entry wound was a reddish-brown abrasion collar, indicative of an entry wound, measuring 2 cm × 2 cm, which was present around the entry wound, indicating that the entry wound was a contact wound. Additionally, a bluish contusion was observed surrounding the entry wound. No exit wound was identified during the examination.

The presence of a reddish-brown abrasion collar surrounding the circular entry wound and the searing around the entry wound indicates that the wound was an entry wound and a contact wound. These findings are consistent with a close-range gunshot injury, further supporting the conclusion of a self-inflicted gunshot wound to the abdomen in this case.

**Figure 3:**
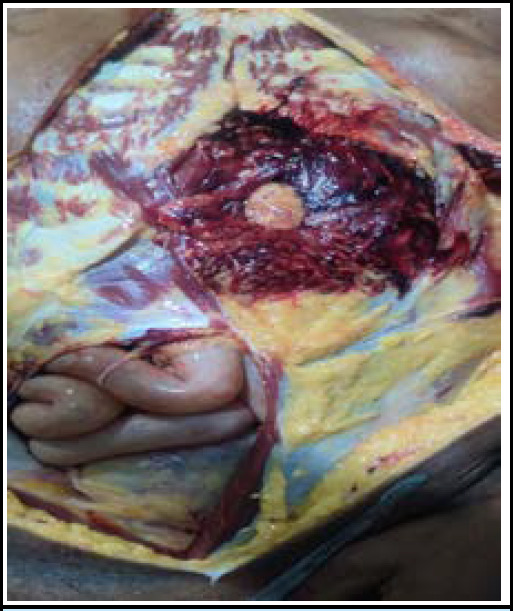
A perforating wound over the peritoneum and mesentery with a surrounding hematoma.

**Figure 4:**
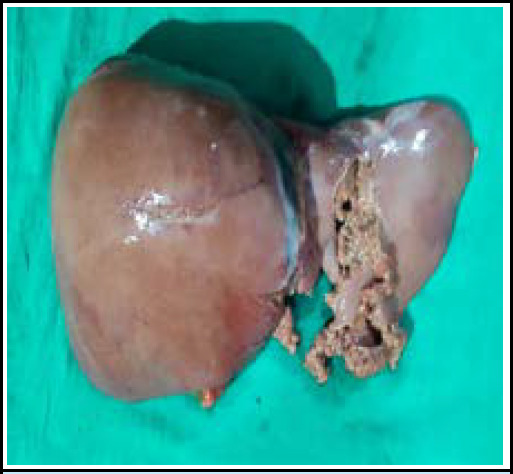
Pulverized left lobe of liver.

**Figure 5:**
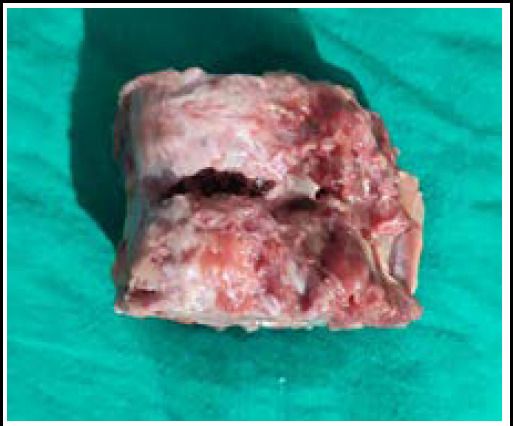
Gutter-like fracture over the T11 vertebra.

During the internal examination, a perforating wound was noted over the peritoneum and mesentery with a surrounding hematoma, consistent with the trajectory of a projectile entering the abdomen (Figure 3). The left lobe of the liver was found to be pulverized, indicating significant damage likely caused by the projectile's path (Figure 4). Additionally, a gutter-like fracture was observed over the T11 vertebra, suggesting the forceful impact of the projectile (Figure 5). The projectile was located over the left lateral aspect of the L1 vertebra, further confirming its path through the body.

## DISCUSSION

Radiological imaging is essential for determining projectile's presence, location, and trajectory within the body. Bullet trajectory analysis plays a crucial role in reenacting a crime scene, aids investigators in understanding what happened, and supplies proof for a suspect's trial.^5^ In this case, the abdominal X-ray confirmed the presence of a metallic projectile within the abdomen, consistent with the clinical findings of an entry wound. The location of the projectile, its deformation, and the surrounding tissue damage provided valuable information regarding the projectile's trajectory.

In a case involving penetrating abdominal trauma, the cooperation between surgeons, radiologists, and forensic pathologists is crucial for a comprehensive understanding of the injury pattern and the factors influencing it.^6^ Radiological imaging,^7^ combined with meticulous autopsy examination, can provide a detailed reconstruction of the event, which is essential for a thorough forensic investigation. CT and MRI imaging can provide additional information on the bullet's path, as well as any potential hazards posed by the retained projectile. However, conventional radiography remains the go-to initial imaging modality due to its widespread availability, ease of use, and relatively lower cost.^6^ Furthermore, radiography can help avoid unnecessary radiation exposure from advanced imaging in cases where the bullet's location is clearly identified. Comprehensive autopsy examination, including both external and internal findings, is essential for accurately determining the nature and mechanism of injury in firearm-related deaths.^7^ In this case, the autopsy findings, such as the characteristics of the entry wound, surrounding abrasion collar, searing, and internal injuries, were consistent with a close-range, self-inflicted gunshot wound to the abdomen.

In this case, the abdominal X-ray with an anterior-posterior view showed a hyper-dense area on the upper left side of the abdomen, resembling the shape of the nose of the bullet, a clear indication of the presence of a metallic projectile.^6^ Moreover, the alignment of the hyper-dense area with the entry wound observed on clinical examination provided additional confirmation of the projectile's trajectory. Shape, size, muzzle velocity and the path taken by projectile after impacting the body affect the amount of tissue damage caused by gunshot wounds.^7^ The absence of significant fragmentation or secondary projectiles indicated a single, direct trajectory consistent with the clinical presentation.

The pulverization of the left lobe of the liver was attributed to the temporary and permanent cavity created by the projectile's kinetic energy. This mechanism of injury resulted in extensive tissue damage along the projectile's path. Furthermore, the location of the projectile over the left lateral aspect of the L1 vertebra was noted to be deviated from its original trajectory. This deviation was likely caused by the projectile's ricocheting from the vertebrae, indicative of the dynamic nature of the injury.

The post-mortem autopsy examination provided further details on the nature and extent of the injury. The external examination revealed a circular entry wound with characteristic features, including an abrasion collar, searing, and a surrounding contusion, suggesting a close-range contact gunshot wound.^8, 9^ The internal examination confirmed the perforating trajectory of the projectile, with significant damage to the left lobe of the liver and a gutter-like fracture of the T11 vertebra, consistent with the projectile's path. The location of the retained projectile over the left lateral aspect of the L1 vertebra, diverged from the original trajectory, suggesting a possible ricochet or deflection of the projectile within the body.

Projectiles enter the body cavity and travel through the tissue with the least resistance.^8^ In this case, the projectile's path through the abdomen, liver, and vertebrae demonstrated the dynamic nature of such injuries, where the bullet trajectory may be altered by anatomical structures, leading to unpredictable and complex injury patterns. Therefore, comprehensive radiological and autopsy examinations are crucial for accurately documenting and interpreting the trajectory and extent of injury in firearm-related cases.

Internal ricochet of the bullet can occur if it strikes hard tissue.^9^ Consistent with our case, the projectile's terminal position may differ from the entry wound due to tracking or deflection within body, possibly requiring additional imaging for accurate diagnosis.^10^ and this evaluation has both clinical relevance (assessment of organ damage, surgical planning, and prognostication Furthermore, one must look for an exit wound if the available imaging does not reveal the presence of a projectile.^10^ and this evaluation has both clinical relevance (assessment of organ damage, surgical planning, and prognostication Hence, X-ray also helps to differentiate between penetrating and perforating wounds.^11^ The management plan mostly depends on the location of retained thoracic projectiles.^12^

Since X-rays provide 2D images and internal tissue destruction cannot be visualized, Magnetic Resonance Imaging (MRI) can appraise of soft tissue injuries better, but metallic bullets that may interact with a high magnetic field are subject to certain limitations.^13^ In contrast to MRI, X-ray and Post Mortem Computed Tomography (PMCT) use ionizing radiation instead of magnetic field, which proves to be more justifiable in case of penetrating trauma by metallic projectiles. Furthermore, as PMCT provides precise and high resolution 3D image in digital format, it can be more reliable imaging tool before forensic autopsy.^14^ which can be collected under the term "post-mortem imaging". Most methods of forensic imaging are from the radiology field and are therefore techniques that show the interior of the body with technologies such as X-ray or magnetic resonance imaging. To digitally image the surface of the body, other techniques are regularly applied, e.g. three-dimensional (3D Comprehensive interpretation of radiological imaging findings in correlation with autopsy results and forensic history is essential to determine the exact trajectory and nature of gunshot wound in medicolegal cases.

Though advanced imaging techniques like Magnetic Resonance Imaging (MRI) and Post Mortem Computed Tomography (PMCT) provide more explicit 3D images, in cases where MRI and PMCT are not easily accessible for the reasons such as technological limitations, budgetary restrictions, or local policies, use of X-rays remains inevitable for investigations prior to autopsy which is quick, easy, and economical to complete.^15^

In developing countries like Nepal, where resources and facilities for comprehensive forensic investigations may be limited, the role of X-ray in autopsies, particularly in cases of firearm injuries, cannot be overstated as it provides crucial information regarding the location and the extent of injury, and potential complications. Despite challenges, the integration of radiological imaging into autopsy procedures can significantly enhance the accuracy and depth of forensic examinations, especially in cases involving penetrating trauma.
